# A review of the 10/66 dementia research group

**DOI:** 10.1007/s00127-018-1626-7

**Published:** 2018-11-22

**Authors:** A. Matthew Prina, Rosie Mayston, Yu-Tzu Wu, Martin Prince

**Affiliations:** Institute of Psychiatry, Psychology & Neuroscience, Department of Health Service, King’s College London, London, UK

**Keywords:** Dementia, LMIC, Epidemiology, Mental health, Ageing

## Abstract

**Background:**

In this review we discuss how the study of dementia epidemiology in Low- and Middle-Income Countries (LMICs) has changed in the last 20 years, and specifically to review the evidence created by the 10/66 Dementia Research Group (DRG) and discuss future directions for research.

**Methods:**

We identified and collated all the papers related to the 10/66 Dementia Research Group, including papers from groups who adopted the 10/66 methodology, that have been published in peer-reviewed journals.

**Results:**

Over 200 papers including data from Africa, Asia, Europe and Latin America and the Caribbean were identified by this review. Many of the findings revolved around the epidemiology of dementia, mental health and non-communicable diseases, including the cross-cultural development and validation of measurement tools of cognition and functioning, need for care, care arrangements and mental health. Social ageing, care dependence and caregiver interventions were also topics that the group had published on.

**Discussion:**

A body of evidence has been generated that has challenged the view, prevalent when the group started, that dementia is comparatively rare in LMICs. The experience of the 10/66 DRG has shown that descriptive epidemiological research can be important and impactful, where few data exist. Monitoring population trends in the prevalence and incidence of dementia may be our best chance to confirm hypotheses regarding modifiable risk factors of dementia.

## Introduction

Population ageing is an important issue across the globe. According to estimates from the United Nations, the number of people aged 60 or above is 962 million in 2017 and over two-thirds lived in low- and middle-income countries (LMICs) [1]. This figure is expected to increase to 2.1 billion by 2050, with nearly 80% living in LMICs. Although pension reform and the introduction of health insurance in some LMICs represent an important step forward, health and social services remain ill-equipped to meet the needs of rapidly growing populations of older people. In recent years, governments of high-income countries (HICs) have had to develop and adopt policies to address the demographic reality of their populations. In many of these settings, there has been increased recognition of the burden and impact of dementia, a syndrome of decline in cognitive function such as memory, language and executive function, characterised by a high degree of disability and dependence, which is estimated to affect 46.8 million people worldwide [2]. In 2013, a G8 summit was held: “Global action against dementia” which resulted in commitment among G8 ministers to increased funding for dementia research, particularly around prevention and treatment, with the key goal of finding a cure or disease-modifying therapy by 2025 [3].

The epidemiological evidence on dementia has been slowly growing for the last 40 years, when to address concerns on dementia in ageing populations, some countries in western Europe and North America started the first epidemiological investigations of dementia [4–8]. Cohort studies with a specific focus on cognitive ageing were set up to investigate dementia in the general population and provide nationwide or regional representative estimates on prevalence and incidence [9].

Early studies from LMICs suggested that dementia was uncommon outside of North America, Western Europe and Australasia. For example, in a community-based study carried out among 932 older Nigerians, no cases of dementia were identified [10]. At the time, authors suggested that environmental risk factors, present in industrialised settings, were likely to be the cause of the observed difference in dementia prevalence. A Delphi consensus study in the early 2000s used expert opinion as the basis for more realistic projections of regional and global prevalence [11]. Although possible regional variations were suggested in this report [11], the epidemiology of dementia in LMICs remained unclear without comparable data across regions. There was a need to obtain empirical evidence from primary research, from representative samples of older people living in community-based settings and using appropriate assessment methods to examine cognitive function and health status, taking into account potential variation in language, culture and research contexts [12].

## 10/66 Dementia research group

The 10/66 Dementia Research Group was formed in 1998, building a network of 30 research groups in 20 countries in Latin America, the Caribbean, India, Russia, China and South East Asia [13], with the explicit goal of generating high-quality research evidence about dementia in LMIC. The name 10/66 reflects the fact that at inception, even though 66% of people with dementia were estimated to live in LMICs, only 10% of the research on dementia was carried out in these settings.

Pilot studies to validate and calibrate a culture and education fair diagnosis of dementia in people aged 65 and over were carried out in 16 low- and middle-income countries across 26 centres between 1999 and 2001. This was followed by a baseline phase of the study, conducted between 2004 and 2006 (see cohort profile [13] for more details). The baseline study was carried out in the following urban and rural sites: Cuba (sites in Havana and Matanzas—both urban), Dominican Republic (Santo Domingo), Puerto Rico (Bayamon), Venezuela (Caracas), Peru (Lima—urban and Canete Province—rural), Mexico (Mexico City—urban and Morelos State—rural), China (Xicheng—urban and Daxing, Beijing province—rural) and India (Chennai—urban and Vellore—rural).

A follow-up wave, including all traceable participants of the baseline assessment was carried out between 2007 and 2010 in the same sites as above, with the exception of India where full follow-up was only available for the mortality outcome or for people identified with cognitive impairment and/ or dementia at baseline. Finally, a third assessment, which includes a new prevalence survey, is currently underway (LIFE2YEARS1066: ERC-2013-ADG [13]). This will include a reassessment of existing participant, and a new prevalence sweep.

The aim of this review is to summarise how the study of dementia and ageing epidemiology in low- and middle-income countries has changed in the last 20 years, and specifically to: (1) collate and review all the evidence created by the 10/66 Dementia Research Group (DRG); (2) summarise this evidence according to themes; (3) identify the broader impact of the 10/66 DRG beyond research; (4) discuss future directions for research on dementia in LMIC.

## Methods

In June 2018 we identified and collated all the papers related to the 10/66 Dementia Research Group that have been published in peer-reviewed journals. We limited our search to papers published in English, which means that some papers published in local languages may have been missed out. A data extraction form was created and infographics created to highlight: (1) time trends of 10/66 publications; (2) geographical distribution of studies. Key findings from each of the thematic areas are presented narratively in the “Results” section.

## Results

**Fig. 1:**
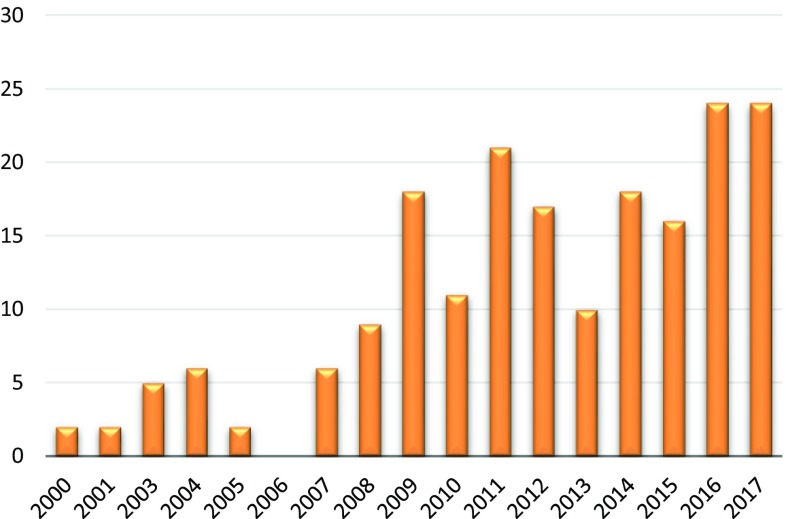
Number of 10/66 related papers by year of publication

**Fig. 2:**
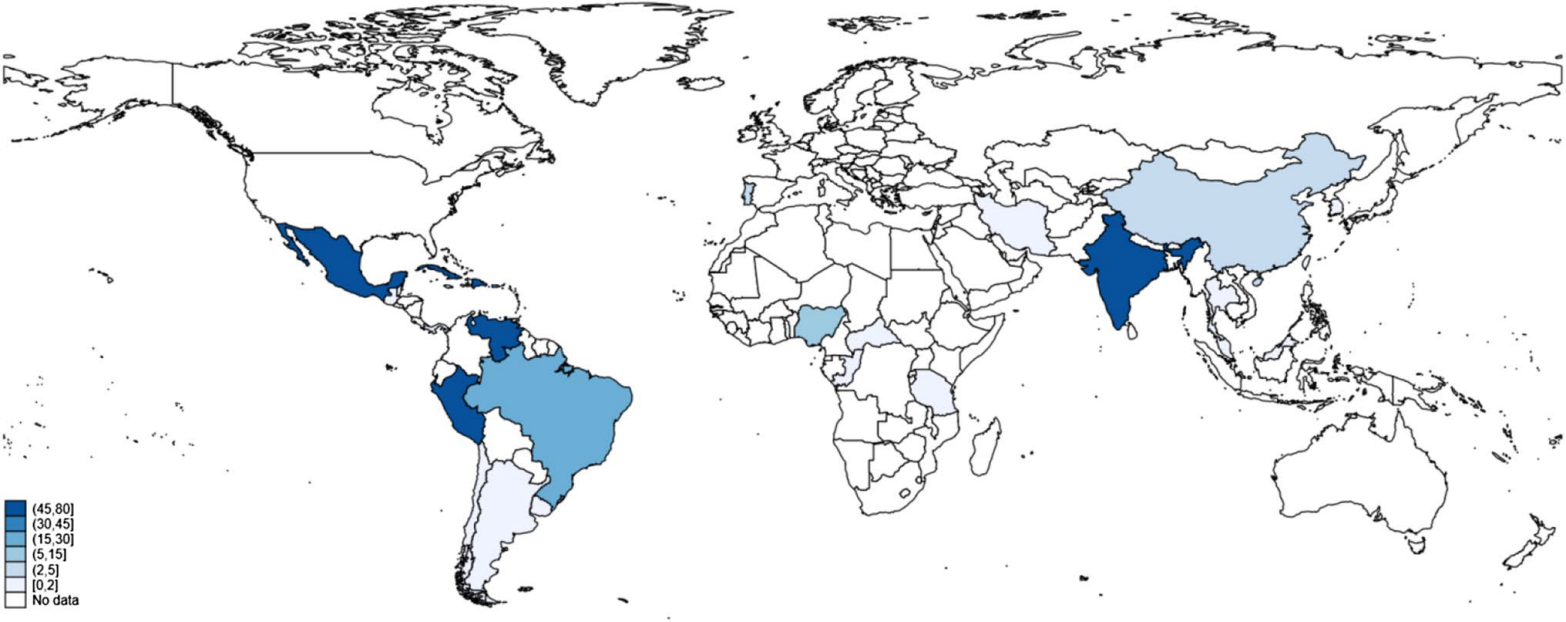
Geographical distribution of 10/66 publications

Over 200 10/66 DRG-related papers have been published since the group’s inception in 1998: 109 on data findings directly carried out by the 10/66 DRG, 51 papers by other groups who used the 10/66 methodology, 21 methodological papers, 17 reviews, and 3 intervention studies. During the first few years, the outputs were limited and focused on position papers, pilot studies and methodological developments, because all the fieldwork was taking place in the sites. 2007 and 2008, saw a flurry of publications, including the publication of the protocols of the population-based research programme [14] and the first comparable estimates of dementia in Latin America, India and China [15]. Since then, outputs have increased on a yearly basis (Fig. 1), expanding beyond the initial aims of the collaboration to address multiple facets of ageing in LMICs, including non-communicable diseases other than dementia, social and economic aspects of ageing. Although the majority of 10/66 publications have come from the core sites (i.e. India, Mexico, Cuba, Peru, Venezuela, Dominican Republic and Puerto Rico), the wider influence of the 10/66 DRG is evidenced by authors who have adopted 10/66 dementia research protocols and dementia diagnostic criteria in other settings (Fig. 2) [16–22].

In the next sections we will summarise the key findings according to each thematic area.

### Method development

A fundamental component of assessing dementia in epidemiological studies lies in its measurement. At the time of the inception of 10/66 DRG, cognitive screening tools developed in high-income countries were shown to be biased by levels education, literacy and numeracy. These instruments were therefore unsuitable for use in LMICs, where older people often had lower levels of education and as a result were less literate and numerate and less able to perform well on cognitive tests, regardless of their cognitive capabilities. During the first few years of the 10/66 DRG, the development of a robust, single-stage diagnostic algorithm with demonstrable validity across different cultural settings and among people with different levels of education, was therefore a key goal.

The 10/66 algorithm was built using existing culture-fair instruments; the CERAD 10-word list recall task, the Community Screening Instrument for Dementia (CSI-D), and the Geriatric Mental State examination (GMS) [14, 23]. The original piloting of this assessment and algorithm was first developed in 10/66 pilot sites across 25 international centres [12]. Norms for the cognitive tests were also developed, indicating a small effect of age, education and culture on the CSI-D COGSCORE and the 10-word delayed recall tests [24]. 10/66 dementia diagnoses were also found to have better agreement with clinical diagnoses in Cuba, compared to DSM-IV computerized diagnoses [15], and better predictive validity in India [25].

More recently, a shorter assessment, which does not include the full Geriatric Mental State interview (GMS) but only the Euro-D depression screen, administered with CSI-D and the CERAD-10 word list recall task was tested across the different 10/66 sites [26]. This showed very good sensitivity and only a small loss of specificity in people with depression, providing an alternative assessment for settings where the GMS interview is not feasible (e.g. lack of time, lack of interviewer training, etc.).

Evidence on the construct, concurrent and predictive validity of other measures in LMICs, including depression measured by EURO-D [27–29], disability [30] and frailty [31] have also been strengthened.

Although the core of the methodological development has been around the psychometric properties of tools and the development of standard operating procedures, the data generated by the group have also been used in other large projects that have tried to harmonise cohort studies of older adults, such as the ATHLOS project (http://athlosproject.eu/).

### Dementia epidemiology

**Fig. 3:**
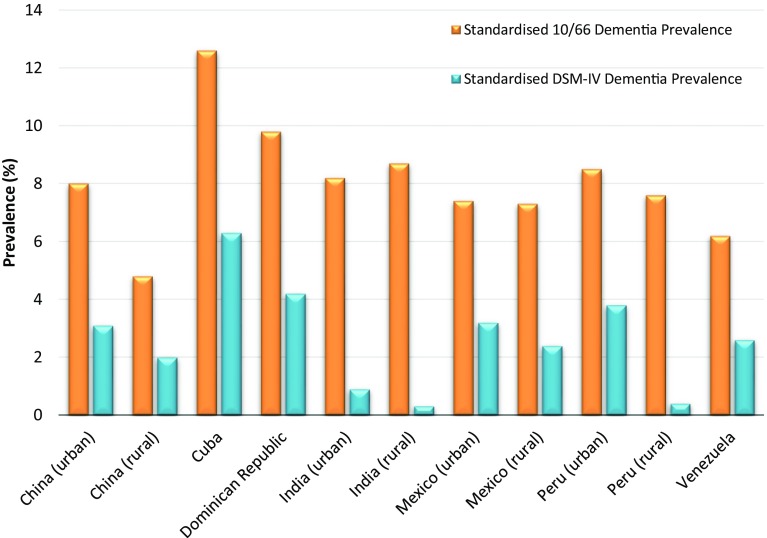
Standardised dementia prevalence across the 10/66 sites. **Standardised for age, sex and education [15]

The prevalence of dementia varied widely across the different 10/66 sites (Fig. 3 [15]), ranging from 4.8% in rural China to 12.6% in Cuba. The wide variation was thought to be the result of the fact that informants in least developed centres were less prone to report social impairment and cognitive decline, which are essential criteria for DSM-IV diagnosis, compared to informants in more developed centres (see section on care dependence below for possible explanations for this finding). This was the case even when an objective memory impairment was identified. It was also concluded that DSM-IV dementia may underestimate the prevalence of dementia, especially in areas with low awareness of dementia, and that the 10/66 prevalence estimates were more consistent with the ‘Global Prevalence of Dementia’ expert consensus that was carried out in 2005 [11].

The incidence of 10/66 dementia varied between 18.2 (95% CI 14.3–23.0) per 1000 person-years in Peru to 30.4 (25.5–36.3) per 1000 person-years in Mexico. This was once again 1.4–2.7 times higher than the incidence of DSM-IV dementia [32]. Being male, having higher education and younger age were all protective of dementia. Specific cognitive tests including literacy, verbal fluency and motor sequencing had also similar protective associations with dementia, providing more supporting evidence for the cognitive reserve hypothesis [32, 33].

In 2013, the DSM-V was introduced, replacing the terms of ‘cognitive disorder not otherwise specified’ and ‘dementia’ with ‘Mild’ and ‘Major’ neurocognitive disorder [34]. The impact of DMS-V dementia criteria in LMICs has not been widely tested, but a small study carried out in the 10/66 Indian site of Chennai showed that although DSM-V case detection is better than DSM-IV, its validity is not robust [35]. Further research is needed to assess the cross-cultural properties of the DMS-V.

### Mental health

An additional key focus of the 10/66 Dementia Research Group has been to generate evidence on the prevalence, impact and risk factors of common mental disorders among older adults living in LMICs. Evidence on the prevalence of anxiety and depressive disorders among older adults living in low- and middle-income countries was also very limited. Findings from the 10/66 baseline, estimated a prevalence of ICD-10 and EURO-D depression with a wide range (ICD-10: from 0.3% in Urban China to 13.8%; EURO-D: 1.0% in rural China to 38.6% in rural India). Although this is quite a large difference between the two diagnostic criteria and across sites, it is consistent with other studies carried out in HIC [36]. The Euro-D identified more cases of depression than the ICD-10, and this is likely to be the result that the latter relies too much upon clinical diagnoses, potentially underestimating the true burden of depression, which can still be very severe in older adults with “sub-syndromal” depression [37].

The prevalence of anxiety was similar to ICD-10 depression estimates [38]. The main anomaly to these estimates lies in China, where low prevalence of both anxiety and depression was reported. Some explanations as to why this is the case are discussed in the original papers, but this is probably the result of contextual factors, which could have affected the ascertainment of common mental disorders in this country [38, 39].

Correlates of anxiety were identified in age, gender, socioeconomic status and number of physical impairments. A history of depression and once again number of physical impairments were the two most consistent correlates of ICD-10 depression across sites [38].

### Non-communicable diseases

Beyond dementia and mental health problems, 10/66 study findings represent an important contribution to a small but growing evidence base on the health of older people. The shift in LMICs towards a burden of disease dominated by non-communicable conditions is reflected in study findings: hypertension was the most common diagnosis (63.6% overall), followed by frailty (15.2%), dementia (8.7%), stroke (7.1%), depression (4.7%) and ischaemic heart disease (4.4%) [40]. Verbal autopsy data showed that the biggest identifiable contributor to deaths was stroke (ascertained as the cause of death for 21.4% of older participants who died during follow-up). Although in some LMICs, some risk factors (such those associated with cardiometabolic disease) have been found to be associated with affluence, 10/66 findings on risk factors for mortality are consistent with a hypothesised link with socioeconomic disadvantage, accumulated across the life-course [41].

Changes in disease burden and patterns of disease burden can be expected to result in increased years lived with disability among older populations. Unlike earlier Global Burden of Disease estimates, which sought to estimate years lost to disability by applying weights to individual diseases, 10/66 estimates were based upon the relative independent contributions of different conditions to overall disability, measured directly using WHODAS-II, an instrument designed to capture the consequences of any health problem; thereby providing a more accurate reflection of the lived experience of older disabled people and their caregivers [30, 42]. Associations with chronic disease accounted for around two-thirds of disability identified in the 10/66 baseline [42].

### Social ageing and care dependence

Consistent with meta-analysis of results from studies carried out in HIC, analysis of 10/66 data has shown that a restricted social network with few friends or community contacts outside the family is associated with higher mortality, as compared to a more integrated social network [43, 44]. These family-dependent or private networks were present across all sites (ranging from 12 to 73% of older participants) [43]. Social support was found to be important in reducing the association between physical impairment and depression in a study carried out in rural Thailand using the Euro-D [45].

Between 3 and 16% of older participants across 10/66 sites were found to have needs for care [42]. After standardising for chronic disease, prevalence of dependence was lower in rural sites in Latin America, China and in rural and urban sites in India; this suggested that perhaps, in less developed settings, where the support of older people is routinely integrated into social roles, particularly of female family members, care dependence may be difficult to separate from normal provision of support to older people [40]. This idea was supported by recent qualitative work carried out in Ghana which suggested that compound living supported the absorption and acceptance of the care of older people into the female role with fewer difficulties than observed in 10/66 Latin American sites [46].

Although in middle-income countries paying for a carer for an older person with care needs is becoming more common (33% in the 10/66 catchment in Lima, 23% in Lebanon), care for dependent older people is largely organised, funded and delivered by family members [47, 48]. 10/66 prevalence studies replicated findings from elsewhere that caregiving was associated with considerable psychological strain [49, 50]. In Lebanon, strain was greatest among carers who provided hands-on care [47]. In 10/66 sites, the norm was for care-dependent older people to live with adult children or children-in-law, women were doing much of the work of caring and carers often reported giving up or cutting back on work to provide care [51].

The INDEP study was designed to investigate earlier 10/66 findings in more depth across four sites: using mixed methods to explore the social and economic effects of caring for dependent older relatives, comparing outcomes among households with older residents needing care with those with independent older residents [48]. Results demonstrated that managing older people’s needs for care had adverse effects upon household economies, including lower paid income, lower consumption and increased healthcare expenditure (including more frequent catastrophic healthcare spending) among care households [52]. Qualitative results revealed the enduring intergenerational financial connectedness of households against a backdrop of changing demands upon family income and resulting strain: e.g. families would struggle to balance the costs of lengthened education for the youngest generation (and the need for women to work to support this) against the expectation that female family members were de facto carers for the oldest generation [53]. Without effective government support, families pieced together the care of older relatives, a task made all the more challenging by the inherent uncertainty of future demands [53]. As reflected in quantitative findings, this often had serious adverse consequences for the household as a whole, including restriction of healthcare use and food consumption [52, 53].

### Helping Carers to Care: intervention studies

Evidence from a meta-analysis of studies carried out in high-income countries showed that psychosocial interventions were effective in reducing psychological distress among caregivers of people living with dementia, improving caregiver knowledge as well as showing benefits to patient mood. There was a lack of evidence from LMICs [54, 55]. Helping Carers to Care is a flexible, stepped care model, designed for use in LMICs’ health system settings. The key objectives are: to improve the knowledge of family caregivers about dementia, provide emotional support to caregivers, maximise caregiving resources and improve caregiving skills [56]. Non-specialist healthcare workers receive 2 days of manualised training in how to deliver the intervention. The intervention is manualised, designed to be delivered over five sessions of 30 min, divided into three modules [14]. Tested using RCT designs in three settings (India, Russia, Peru), the intervention has demonstrated moderate positive effects upon caregiver mental health (India, Russia) and caregiver strain (Peru), as well as reduction in distress due to problem behaviours (India) [56–58]. Findings are in line with moderate effect sizes identified in caregiver focussed interventions in high-income country settings. Low drop-out across the three sites suggests that this kind of intervention delivered by non-specialists is acceptable to people living with dementia and their families, in these contexts. However, questions remain about the feasibility and scalability of such an intervention without changes to the health system originally designed to be delivered by non-specialist primary healthcare workers.

### Broader impact of the 10/66 DRG work

1. Methodology

The core 10/66 studies were all originally led and coordinated from King’s College London, with 118 peer-reviewed publications to date from the 10/66 pilot studies, the prevalence wave, the incidence wave, and the nested INDEP study of care dependence. Wider impact has been achieved, for academic beneficiaries, by promoting secondary data analysis through facilitated access to our Public Data Archive (https://www.alz.co.uk/1066/1066_public_archive_baseline.php), and through support provided to other research groups to adapt and use our research methods, tailored to their particular needs.

We have supported two national representative surveys sponsored by national governments; the Wellbeing of the Singapore Elderly (WiSE study—working with the Institute of Mental Health, Singapore) [59], and the Trinidad national survey of ageing and cognition (The University of the West Indies) [60]. For the Trinidad survey a briefer version of the 10/66 Dementia Diagnostic algorithm was deployed for the first time, developed and validated on the earlier 10/66 pilot and prevalence wave samples [26]. This reduces considerably training demands, survey time and interview burden by substituting the briefer 12-item EURO-D depression scale for the full Geriatric Mental State clinical interview. The full 10/66 protocol was used to conduct a pilot survey of dementia prevalence in Beirut and Mount Lebanon Governorates (American University of Beirut, University of Copenhagen) [61], and surveys of one urban and one rural catchment area in Portugal (Universidade Nova de Lisboa) [22]. 10/66 Dementia Diagnostic assessments were included in the EPIDEMCA surveys conducted in Central African Republic and Republic of Congo, (University of Limoges, INSERM UMR 1094) [20], and in the rural Hai district of northern Tanzania (Newcastle University) [21]. 10/66 methodologies are also being used to study dementia and cognitive outcomes in the MYsore studies of Natal effects on Ageing and Health (MYNAH—Epidemiology Research Unit, CSI Holdsworth Memorial Hospital and University of Southampton) lifecourse cohort in southern India [62].

These studies have extended considerably the geographic and cultural scope of the evidence provided by core 10/66 studies focused on Latin America, the Hispanic Caribbean, China and India. Additional evidence has been obtained to support the validity of the diagnostic approach; in Lebanon for the Arabic version of the assessment, and in nursing homes as well as community samples [19]; and in Singapore across Malay, Tamil and Chinese sub-populations [59].

2. Policy impact

Findings from the 10/66 Dementia Research Group prevalence and incidence wave studies, and methodologically linked projects have been actively disseminated, forming an important component of the evidence base for LMICs for the series of World Alzheimer reports (2009–2016, on the global prevalence, incidence and costs of dementia, early intervention, long-term care, modifiable risk factors, and health system responses), and the landmark 2012 WHO report “Dementia—a public health priority” (2012). These documents became the standard source on global dementia burden and impact for academic and media reports, and, once adopted by WHO, the basis for the G7 countries Global Action on Dementia, the WHO ‘Call for Action’ [63] and subsequent Global Action Plan on the public health response to dementia (2017–2025) [64].

The key messages that emerged from this process of policymaker engagement were that

1. The age-specific prevalence and incidence of dementia was similar in LMICs to that of HICs.
2. Given the distribution of world’s older population, and projected rates of demographic ageing, nearly two-thirds of people with dementia reside in LMIC, with numbers expected to rise much more rapidly in those regions.
3. Rates of contact coverage for health and social care were low everywhere, but negligible in LMICs.
4. The lack of a disease-modifying treatment, timely diagnosis and access to advice symptomatic treatment, and support should be made generally available through a public health-orientated model of task-shifted care, supported where feasible by specialist providers.

Evidence on case-finding and detection, and caregiver intervention was included in the development of WHO mhGAP guidelines for the assessment and management of dementia, and the WHO I-COPE ‘Integrated Care for Older People’ guideline.

## Discussions

The 10/66 DRG experience has shown that descriptive epidemiological research can be important and impactful, where few data exist. When the group began its activity in the late 1990s, it was estimated that less than 10% of population-based research into dementia had been conducted in LMIC, where up to two-thirds of those affected might live. A body of evidence has accumulated challenging the view, prevalent at that time, that the condition was comparatively rare in those regions. 20 years on, after an upsurge of studies in LMICs, and a precipitous decline in research in HICs, the overwhelming majority of descriptive studies in recent studies have emanated from LMICs [2]. The notion that evidence, once obtained, can be applied in perpetuity is obviously fallacious. Secular trends (that is, gradual decreases or increases in prevalence over long-term periods) are perfectly plausible. These may be driven by changes in incidence, or survival with dementia, or both. There is currently some tentative evidence that incidence rates may be declining in some high-income countries [7, 65], consistent with increasing education levels and improvements in cardiovascular health; few studies have applied consistent methodology to the repeated assessment of the same population over time, and none to date in LMIC, where trends in cardiovascular health have been proceeding in an adverse direction [66]. This is one of the main objectives for the current third wave of 10/66 DRG population-based studies (LIFE2YEARS 10 years on) [13]. Monitoring population trends in the prevalence and incidence of dementia may be our best chance to confirm hypotheses regarding modifiable risk factors [67], and is essential for future policymaking and planning with respect to provision of health services and long-term care. The assessment of diagnostic and contact coverage (for basic community services), care arrangements and support for informal caregivers, and societal costs and their distribution are all core indicators for the WHO Global Dementia Observatory (GDO). The GDO was established to measure progress on implementation of the 2017–2025 Global Dementia Action Plan, and assist member states in strengthening policies, and dementia health and social care system planning [68].

Cardiovascular disease and cancer are the leading contributors to disease burden among older people in all world regions, mainly because of their impact on mortality [69]. However, dementia (alongside other conditions of the brain and mind such as stroke and depression) is the leading independent contributor to disability and care dependence [40, 42]. Older people frequently live with multiple physical, mental and cognitive disorders. Frailty and multimorbidity are important concepts for summarising individual and population health, and quality of life, disability, and care dependence are the key outcomes with respect to impact on the older person, their family and carers, and wider society. The 10/66 DRG surveys were, from the outset fairly compendious studies of health and ageing, with an emphasis on social context and impact. With the exception of cancer, all common chronic conditions are assessed. Later waves of the survey were extended to include more comprehensive coverage of physical frailty indicators. Economic circumstances, unpaid and paid care arrangements, service utilisation and out-of-pocket costs have been assessed throughout, while later waves of the survey have included household consumption and economic strain indicators, and additional information on co-residents. The yield from such studies is far greater and more informative than that from traditional dementia prevalence studies. This is clearly demonstrated by the Singapore WiSE study, which used the full 10/66 survey protocol, and has largely followed its research agenda, with 34 publications. In many of these respects, the 10/66 DRG studies resemble the US National Institute of Aging sponsored Health and Retirement Study (HRS) family of studies most of which include nationally representative samples. These were extended from the USA to European countries (SHARE, ELSA and TILDA), Japan (JSTAR), South Korea (KLoSA), and several LMIC; China, Ghana, India, Mexico, Russia and South Africa (SAGE), Mexico (MHAS), India (LASI), China (CHARLS), and Indonesia (IFLS). While all of these initiatives are welcome, ultimately it will be the responsibility of governments, and very much in their interests, to monitor the health and wellbeing of their older populations through comprehensive national representative health and demographic surveillance studies. Mainstreaming of cognitive health and dementia diagnostic indicators within such surveys would indicate that appropriate priority is being accorded to the promotion of cognitive health, the prevention of cognitive disorder, and the care of people with dementia, and support of their carers. A formal survey diagnosis of dementia may not be necessary for these purposes. Ultimately the most important legacy of the 10/66 DRG program may be the cross-cultural development and validation of brief assessments of cognition and functioning (that can be mapped probabilistically on to dementia diagnosis), needs for care, care arrangements and their impact.

Work conducted by the 10/66 DRG for example on the burden and impact of physical comorbidity in dementia [70], the effectiveness of caregiver intervention, community case-finding, the development of brief screening tools [71], and holistic assessment approaches [72] has informed the development of current WHO clinical guidelines for dementia (mhGAP) and Integrated Care for Older People (ICOPE). Implementation of more vertical (mhGAP) or horizontal (ICOPE) approaches to task-shifted detection, assessment and management may depend upon health-system preparedness. In either case, as with monitoring the health and wellbeing of the older population, an integrated and holistic approach is likely to be most effective.
